# Supplementary material for: Immunopeptidomic analysis of influenza A virus infected human tissues identifies internal proteins as a rich source of HLA ligands
Source: PLoS Pathog. 2022 Jan 20;18(1):e1009894. doi: 10.1371/journal.ppat.1009894 (PMC8806059; doi:10.1371/journal.ppat.1009894)
Supplement: S1 Table — (PDF) [file ppat.1009894.s001.pdf]

S1 Table: HLA allotypes of the cell lines, lung tissues and dendritic cells used in the study

ID	1	2	3	7	8	9	4	5	6	10	11	12
THP1	A*02:01	B*15:11	C*03:03	A*02:01	B*15:11	C*03:03	DRB1*01:01	DQB1*05:01	DPB1*02:01	DRB1*15:01	DQB1*06:02:	DPB1*04:02
P1	A*03:01	B*07:02	C*07:02	A*26:01	B*07:02	C*07:02	DRB1*11:01	DQB1*03:01	DPB1*02:01	DRB1*15:01	DQB1*06:02	DPB1*16:01
P2	A*01:01	B*15:01	C*03:03	A*02:01	B*44:27	C*07:04	DRB1*04:01	DQB1*03:02	DPB1*03:01	DRB1*15:01	DQB1*06:03	DPB1*04:01
P3	A*02:01	B*15:01	C*03:04	A*03:01	B*18:01	C*05:01	DRB1*11:01	DQB1*03:01	DPB1*01:01	DRB1*15:01	DQB1*06:02	DPB1*04:01
P4	A*01:01	B*51:01	C*06:02	A*01:01	B*57:01	C*07:01	DRB1*04:03	DQB1*03:03	DPB1*04:01	DRB1*07:01	DQB1*03:05	DPB1*14:01
A549	A*25:01	B*18:01	C*12:03	A*30:01	B*44:03	C*16:01	DRB1*07:01	DQB1*02:02	DPB1*03:01	DRB1*11:04	DQB1*03:01	DPB1*06:01
