# Supplementary material for: Immunopeptidomic analysis of influenza A virus infected human tissues identifies internal proteins as a rich source of HLA ligands
Source: PLoS Pathog. 2022 Jan 20;18(1):e1009894. doi: 10.1371/journal.ppat.1009894 (PMC8806059; doi:10.1371/journal.ppat.1009894)
Supplement: S2 Table — RsPa = resected lung parenchyma tissue, MoDC = monocyte-derived dendritic cells differentiated in vitro from PBMCs from donor P4. (PDF) [file ppat.1009894.s002.pdf]

S2 Table: Summary of immunopeptidomes isolated from cell lines, lung tissues and dendritic cells. *RsPa = resected lung parenchyma tissue, MoDC = monocyte-derived dendritic cells differentiated in vitro from PBMCs from donor P4.*

ID	Sample type	No. peptides	No. proteins	Strain	HLA
THP1	Cell line	10,709	3,064	Wisconsin	I
THP1	Cell line	11,643	3,308	X-31	I
P1	RsPa	7,944	2,603	Wisconsin	I
P1	RsPa	1,038	324	Wisconsin	II
P2	RsPa	5,304	1,696	Wisconsin	I
P2	RsPa	1,826	438	Wisconsin	II
P2	RsPa	5,985	1,870	X-31	I
P2	RsPa	1,469	369	X-31	II
P3	RsPa	6,338	1,996	Wisconsin	I
P3	RsPa	1,091	327	Wisconsin	II
P3	RsPa	5,926	1,891	X-31	I
P3	RsPa	1,170	341	X-31	II
P4	MoDC+A549	5,432	2,614	Wisconsin	I
P4	MoDC+A549	4,639	891	Wisconsin	II
