# Supplementary material for: Immunopeptidomic analysis of influenza A virus infected human tissues identifies internal proteins as a rich source of HLA ligands
Source: PLoS Pathog. 2022 Jan 20;18(1):e1009894. doi: 10.1371/journal.ppat.1009894 (PMC8806059; doi:10.1371/journal.ppat.1009894)
Supplement: S3 Table — nM is NetMHC 4.0 predicted binding affinity. (PDF) [file ppat.1009894.s003.pdf]

S3 Table: Immunopeptides isolated from A549 cells following infection with A/Wisconsin/67/2005 influenza. *nM is NetMHC 4.0 predicted binding affinity.*

Protein	Protein Position	Length	Allotype	nM	Peptide
Non-structural protein 1	142-150	9	HLA-A*25:01	24	ETIVLLRAF
Nucleoprotein	342-351	10	HLA-A*30:01	538	RLLSFIRGTK
Matrix Protein 1	47-56	10	HLA-A*30:01	22	KTRPILSPLT
