# Supplementary material for: Immunopeptidomic analysis of influenza A virus infected human tissues identifies internal proteins as a rich source of HLA ligands
Source: PLoS Pathog. 2022 Jan 20;18(1):e1009894. doi: 10.1371/journal.ppat.1009894 (PMC8806059; doi:10.1371/journal.ppat.1009894)
Supplement: S4 Table — (PDF) [file ppat.1009894.s004.pdf]

S4 Table: Influenza peptides identified in Immune Epitope Database (2021-11-03) and from immunopeptidome experiments.

IEDB	Peptide	Length	IEDB Allotypes	IEDB Evidence	IEDB Assays	PUBMED ID	Protein	Protein Pos.	Exp. Sample	Exp. Strain	Exp. Allotype	NetMHC nM
IEDB_EPITOPE:2014	AIMDKNIIL	9	HLA-A*02:01, HLA-A*02:02, HLA-A*02:03, HLA-A*02:06, HLA-A2, HLA-A*68:02	Allele/Locus-specific Antibody, Cited reference, Inferred by motif or alleles present, MHC binding assay, Not determined	activation|biological activity, cytotoxicity|51 chromium, cytotoxicity|in vivo assay, decreased disease|in vivo assay, degranulation|biological activity, half maximal inhibitory concentration (IC50)|cellular MHC/competitive/fluorescence|nM, half maximal inhibitory concentration (IC50)|purified MHC/competitive/radioactivity|nM, IFNg release|ELISPOT, IFNg release|ICS, IL-2 release|ICS, ligand presentation|coelution, ligand presentation|secreted MHC/mass spectrometry , qualitative binding|multimer/tetramer, TNF release|ICS	10364497, 11752149, 16731941, 18353950, 18614638, 19734234, 21765016, 23144892, 23146941, 23152369, 23681926, 24257602, 25646416, 26232347, 26509579, 30418433, 7547687, 9647214	Non-structural protein 1	122-130	THP1-X31	X-31	HLA-A*02:01	53.78
IEDB_EPITOPE:2022	AIMEKNIML	9	HLA-A*02:01	Allele/Locus-specific Antibody, MHC binding assay	cytotoxicity|51 chromium, half maximal inhibitory concentration (IC50)|purified MHC/competitive/radioactivity|nM, ligand presentation|secreted MHC/mass spectrometry	7547687	Non-structural protein 1	122-130	THP1-Wis	Wisconsin	HLA-A*02:01	23.35
IEDB_EPITOPE:9163	DLLENLQAY	9	HLA-A*01:01, HLA-A*02:01, HLA-A*03:01, HLA-A*11:01, HLA-A*24:02, HLA-A*26:01, HLA-A3, HLA-A*30:01, HLA-A*31:01, HLA-A*69:01, HLA-B*07:02, HLA-B*08:01, HLA-B*15:01, HLA-B*27:05, HLA-B*35:01, HLA-B*40:01, HLA-B*44:02, HLA-B*58:01, pig	MHC binding prediction	dissociation constant KD (~EC50)|purified MHC/direct/fluorescence|nM, half life|purified MHC/direct/radioactivity|min, IFNg release|ELISPOT, qualitative binding|cellular MHC/direct/fluorescence, qualitative binding|purified MHC/direct/fluorescence, qualitative binding|purified MHC/direct/radioactivity	27411061, 7679507, 7684681	Matrix protein 1	232-240	THP1-X31	X-31	HLA-B*15:11	
IEDB_EPITOPE:9164	DLLENLQTY	9	HLA-A*26:01	NULL	dissociation constant KD (~EC50)|purified MHC/direct/fluorescence|nM, half life|purified MHC/direct/radioactivity|min	NULL	Matrix protein 1	232-240	THP1-Wis	Wisconsin	HLA-B*15:11	

IEDB	Peptide	Length	IEDB Allotypes	IEDB Evidence	IEDB Assays	PUBMED ID	Protein	Protein Pos.	Exp. Sample	Exp. Strain	Exp. Allotype	NetMHC nM
IEDB_EPITOPE:17119	FMYSDFHFI	9	HLA-A*02:01, HLA-A*02:02, HLA-A*02:03, HLA-A*02:06, HLA-A2, HLA-A*24:02, HLA-A*68:02, HLA-A*69:01, HLA-B*15:01, HLA-B*51:01, HLA-C*07:02	Allele/Locus-specific Antibody, Cited reference, Inferred by motif or alleles present, MHC binding assay	activation|biological activity, cytotoxicity|51 chromium, cytotoxicity|in vivo assay, decreased disease|in vivo assay, dissociation constant KD (~EC50)|purified MHC/direct/fluorescence|nM, dissociation constant KD (~IC50)|purified MHC/competitive/radioactivity|nM, dissociation constant KD|purified MHC/competitive/fluorescence|nM, half life|purified MHC/direct/radioactivity|min, half maximal inhibitory concentration (IC50)|purified MHC/competitive/radioactivity|nM, IFNg release|ELISPOT, IFNg release|ICS, ligand presentation|secreted MHC/mass spectrometry , pathogen burden after challenge|in vivo assay, proliferation|in vivo assay, qualitative binding|multimer/tetramer, qualitative binding|purified MHC/competitive/fluorescence, qualitative binding|purified MHC/competitive/radioactivity, survival from challenge|in vivo assay, TNFa release|ICS	10773346, 19734234, 20156506, 21765016, 21846352, 23146941, 23202497, 25311806, 25646416, 26276509, 26509579, 27036003, 27036323, 27333291, 28564390, 29593747, 29895573, 30418433, 31105686	Protein PA-X	46-54	THP1-X31	X-31	HLA-A*02:01	2.66

IEDB	Peptide	Length	IEDB Allotypes	IEDB Evidence	IEDB Assays	PUBMED ID	Protein	Protein Pos.	Exp. Sample	Exp. Strain	Exp. Allotype	NetMHC nM
IEDB_EPITOPE:17119	FMYSDFHFI	9	HLA-A*02:01, HLA-A*02:02, HLA-A*02:03, HLA-A*02:06, HLA-A2, HLA-A*24:02, HLA-A*68:02, HLA-A*69:01, HLA-B*15:01, HLA-B*51:01, HLA-C*07:02	Allele/Locus-specific Antibody, Cited reference, Inferred by motif or alleles present, MHC binding assay	activation|biological activity, cytotoxicity|51 chromium, cytotoxicity|in vivo assay, decreased disease|in vivo assay, dissociation constant KD (~EC50)|purified MHC/direct/fluorescence|nM, dissociation constant KD (~IC50)|purified MHC/competitive/radioactivity|nM, dissociation constant KD|purified MHC/competitive/fluorescence|nM, half life|purified MHC/direct/radioactivity|min, half maximal inhibitory concentration (IC50)|purified MHC/competitive/radioactivity|nM, IFNg release|ELISPOT, IFNg release|ICS, ligand presentation|secreted MHC/mass spectrometry , pathogen burden after challenge|in vivo assay, proliferation|in vivo assay, qualitative binding|multimer/tetramer, qualitative binding|purified MHC/competitive/fluorescence, qualitative binding|purified MHC/competitive/radioactivity, survival from challenge|in vivo assay, TNFa release|ICS	10773346, 19734234, 20156506, 21765016, 21846352, 23146941, 23202497, 25311806, 25646416, 26276509, 26509579, 27036003, 27036323, 27333291, 28564390, 29593747, 29895573, 30418433, 31105686	Protein PA-X	46-54	THP1-X31	X-31	HLA-C*03:03	94.61
IEDB_EPITOPE:17455	FPYTGDPPY	9	HLA-A*26:01, HLA-A*68:23, HLA-B*07:02, HLA-B*15:42, HLA-B*35:01, HLA-B*45:06, HLA-B*51:01, HLA-B*83:01, HLA-C*04:01	NULL	dissociation constant KD (~EC50)|purified MHC/direct/fluorescence|nM, dissociation constant KD|purified MHC/competitive/fluorescence|nM, half life|purified MHC/direct/radioactivity|min	NULL	RNA polymerase catalytic subunit	22-30	THP1-X31	X-31	HLA-B*15:11	
IEDB_EPITOPE:124126	FQNVNKITY	9	HLA-B*15:01, HLA-B*46:01	NULL	dissociation constant KD|purified MHC/competitive/fluorescence|nM, half life|purified MHC/direct/radioactivity|min	NULL	Hemagglutinin	310-318	THP1-X31	X-31	HLA-B*15:11	
IEDB_EPITOPE:124127	FQNVNRITY	9	HLA-B*15:01, HLA-B*35:01	NULL	dissociation constant KD|purified MHC/competitive/fluorescence|nM, half life|purified MHC/direct/radioactivity|min	NULL	Hemagglutinin	310-318	THP1-Wis	Wisconsin	HLA-B*15:11	
IEDB_EPITOPE:28891	ITFHGAKEI	9	HLA-A*02:01	NULL	qualitative binding|cellular MHC/direct/fluorescence	7684681	Matrix protein 1	107-115	THP1-X31	X-31	HLA-B*15:11	
IEDB_EPITOPE:768533	LTFLARSAL	9	SLA-2*11:04	MHC binding prediction	IFNg release|ELISPOT	29772011	Nucleoprotein	256-264	THP1-X31	X-31	HLA-C*03:03	419.04

IEDB	Peptide	Length	IEDB Allotypes	IEDB Evidence	IEDB Assays	PUBMED ID	Protein	Protein Pos.	Exp. Sample	Exp. Strain	Exp. Allotype	NetMHC nM
IEDB_EPITOPE:54584	RLIDFLKDV	9	HLA-A*02:01, HLA-A*02:02, HLA-A*02:03, HLA-A*02:06, HLA-A*68:02	MHC binding assay	cytotoxicity|51 chromium, half life|purified MHC/direct/radioactivity|min, half maximal inhibitory concentration (IC50)|cellular MHC/competitive/fluorescence|nM, half maximal inhibitory concentration (IC50)|purified MHC/competitive/radioactivity|nM	10773346, 19734234	RNA polymerase catalytic subunit	162-170	THP1-X31	X-31	HLA-A*02:01	23.34
IEDB_EPITOPE:54888	RMGAVTTEV	9	HLA-A*02:01, HLA-A*02:02, HLA-A*02:03, HLA-A*02:06, HLA-A*68:02, HLA-DRA*01:01/DRB1*01:01, HLA-DRB1*01:01	Allele/Locus-specific Antibody, MHC binding assay, MHC binding prediction	dissociation constant KD (~EC50)|purified MHC/direct/fluorescence|nM, half maximal inhibitory concentration (IC50)|cellular MHC/competitive/fluorescence|nM, half maximal inhibitory concentration (IC50)|purified MHC/competitive/radioactivity|nM, IFNg release|ELISPOT, ligand presentation|secreted MHC/mass spectrometry , qualitative binding|cellular MHC/direct/fluorescence	19734234, 25646416, 26731261, 7506574, 7684681	Matrix protein 1	134-142	THP1-X31	X-31	HLA-A*02:01	23.50
IEDB_EPITOPE:2022	AIMEKNIML	9	HLA-A*02:01	Allele/Locus-specific Antibody, MHC binding assay	cytotoxicity|51 chromium, half maximal inhibitory concentration (IC50)|purified MHC/competitive/radioactivity|nM, ligand presentation|secreted MHC/mass spectrometry	7547687	Non-structural protein 1	122-130	P2	Wisconsin	HLA-A*02:01	23.35
IEDB_EPITOPE:2022	AIMEKNIML	9	HLA-A*02:01	Allele/Locus-specific Antibody, MHC binding assay	cytotoxicity|51 chromium, half maximal inhibitory concentration (IC50)|purified MHC/competitive/radioactivity|nM, ligand presentation|secreted MHC/mass spectrometry	7547687	Non-structural protein 1	122-130	P3	Wisconsin	HLA-A*02:01	23.35
IEDB_EPITOPE:124082	ETIVLLRAF	9	HLA-A*26:01	NULL	dissociation constant KD|purified MHC/competitive/fluorescence|nM, half life|purified MHC/direct/radioactivity|min	NULL	Non-structural protein 1	142-150	P1	Wisconsin	HLA-A*26:01	18.52
IEDB_EPITOPE:16567	FLEESHPGIF	10	Mamu-A1*002:01	NULL	dissociation constant KD (~IC50)|purified MHC/competitive/radioactivity|nM	NULL	RNA polymerase catalytic subunit	94-103	P2	X-31	HLA-B*44:27	
IEDB_EPITOPE:54656	RLLQNSQVY	9	HLA-A3	NULL	qualitative binding|purified MHC/direct/radioactivity	7679507	Nucleoprotein	305-313	P2	X-31	HLA-B*15:01	37.33
IEDB_EPITOPE:54898	RMGVQMQRFK	10	HLA-A*11:01	MHC binding assay	cytotoxicity|51 chromium, half maximal inhibitory concentration (IC50)|purified MHC/competitive/radioactivity|nM	9366399	Matrix protein 1	243-252	P1	Wisconsin	HLA-A*03:01	23.32

IEDB	Peptide	Length	IEDB Allotypes	IEDB Evidence	IEDB Assays	PUBMED ID	Protein	Protein Pos.	Exp. Sample	Exp. Strain	Exp. Allotype	NetMHC nM
IEDB_EPITOPE:68383	VETPIRNEW	9	H2-Kd, HLA-B*40:01, HLA-B44, HLA-B*44:03	Cited reference, T cell assay - Mismatched MHC molecules	cytotoxicity|51 chromium, dissociation constant KD (~EC50)|purified MHC/direct/fluorescence|nM, IFNg release|ICS, qualitative binding|multimer/tetramer	10358215, 27775159, 34198851, 9765409	Matrix protein 2	7-15	P2	X-31	HLA-B*44:27	
IEDB_EPITOPE:13582	ENRMVLASTTAKAME	15	NULL	NULL	IFNg release|ELISPOT	16140434	Matrix protein 1	176-190	P4	Wisconsin	HLA-DRB1*04:03/07:01	161.63
IEDB_EPITOPE:13582	ENRMVLASTTAKAME	15	NULL	NULL	IFNg release|ELISPOT	16140434	Matrix protein 1	176-190	P4	Wisconsin	HLA-DRB1*04:03/07:01	78.62
IEDB_EPITOPE:128850	HENRMVLASTTAKAMEQ	17	HLA-DR1, HLA-DR4, mouse	MHC binding prediction, T cell assay -T cell subset identification	IFNg release|ELISPOT, IL-2 release|ELISPOT	19524006, 20090904, 22467652	Matrix protein 1	175-191	P4	Wisconsin	HLA-DRB1*04:03/07:01	
IEDB_EPITOPE:606271	HENRMVLASTTAKAMEQM	18	human	T cell assay -T cell subset identification	IFNg release|ICS	28421076, 34198851	Matrix protein 1	175-192	P4	Wisconsin	HLA-DRB1*04:03/07:01	
IEDB_EPITOPE:1309539	NRMVLASTTAKAMEQ	15	HLA-DRB1*04:02	NULL	dissociation constant KD (~IC50)|purified MHC/competitive/fluorescence|nM	32887877	Matrix protein 1	177-191	P4	Wisconsin	HLA-DRB1*04:03/07:01	166.75
IEDB_EPITOPE:1309539	NRMVLASTTAKAMEQ	15	HLA-DRB1*04:02	NULL	dissociation constant KD (~IC50)|purified MHC/competitive/fluorescence|nM	32887877	Matrix protein 1	177-191	P4	Wisconsin	HLA-DRB1*04:03/07:01	77.86
