# Supplementary material for: Immunopeptidomic analysis of influenza A virus infected human tissues identifies internal proteins as a rich source of HLA ligands
Source: PLoS Pathog. 2022 Jan 20;18(1):e1009894. doi: 10.1371/journal.ppat.1009894 (PMC8806059; doi:10.1371/journal.ppat.1009894)
Supplement: S1 Fig — THP1MF were enzymatically dispersed from the culture surface prior to fixation and permeabilization of the cells. Cells were stained intracellularly with FITC-conjugated anti-nucleoprotein antibodies and analysed by flow cytometry. Infected cells were gated with respect to cells exposed to UV-inactivated virus as controls. Figure shown is representative of three independent experiments. (PDF) [file ppat.1009894.s005.pdf]

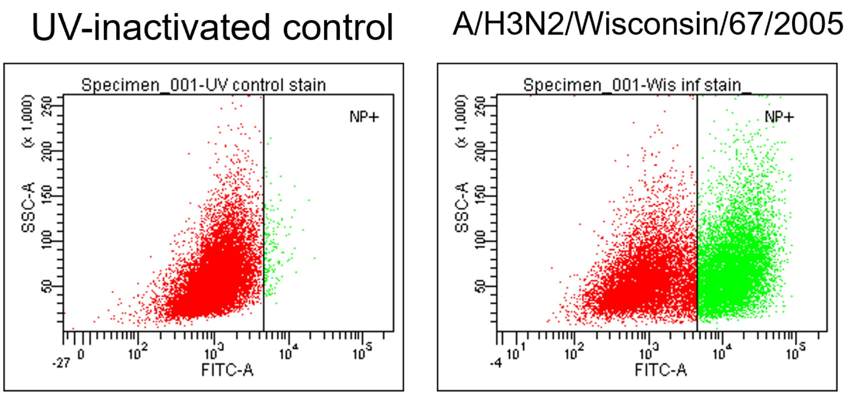

S1 Fig: Flow cytometric identification of infected THP1MF 24 h post-infection. *THP1MF were enzymatically dispersed from the culture surface prior to fixation and permeabilization of the cells. Cells were stained intracellularly with FITC-conjugated anti-nucleoprotein antibodies and analysed by flow cytometry. Infected cells were gated with respect to cells exposed to UV-inactivated virus as controls. Figure shown is representative of three independent experiments.*
