# Supplementary material for: Immunopeptidomic analysis of influenza A virus infected human tissues identifies internal proteins as a rich source of HLA ligands
Source: PLoS Pathog. 2022 Jan 20;18(1):e1009894. doi: 10.1371/journal.ppat.1009894 (PMC8806059; doi:10.1371/journal.ppat.1009894)
Supplement: S2 Fig — Viral proteins were analysed by mass spectrometry proteomics, and the relative quantities of each protein was determined as described in the methods. Quantities are expressed as the percentage of the intensity of the top 3 peptides from each protein from 3 technical replicates. (PDF) [file ppat.1009894.s006.pdf]

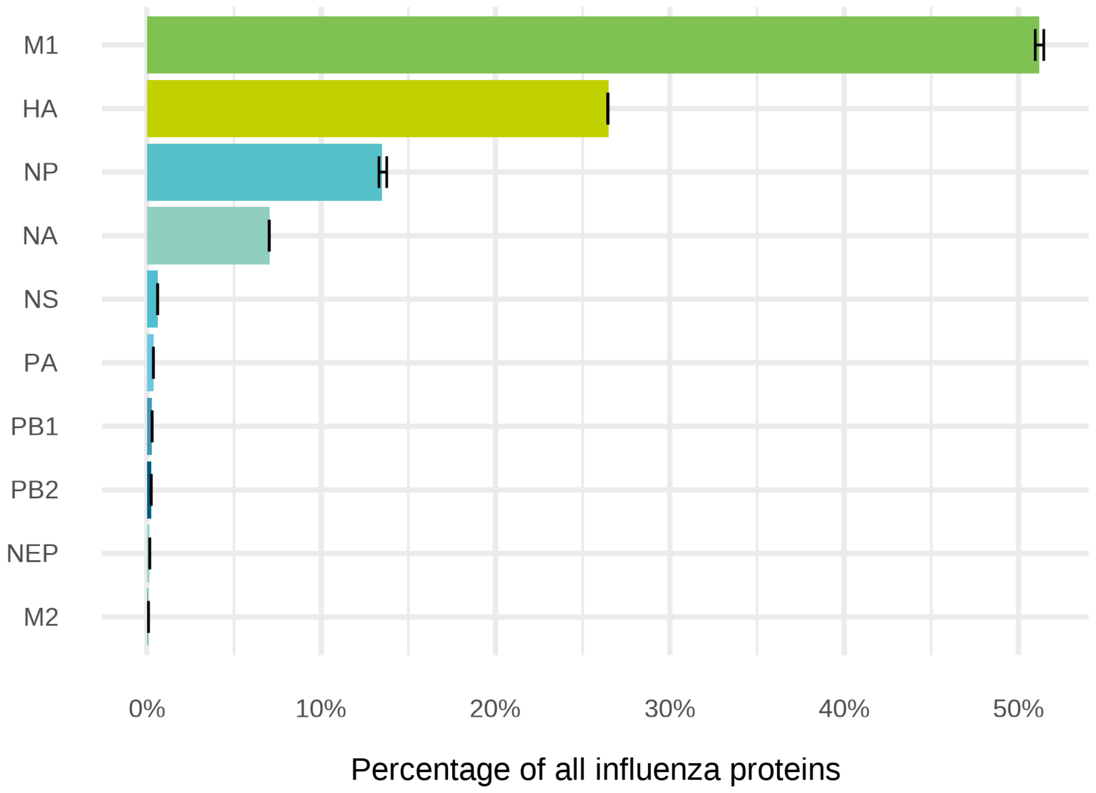

S2 Fig: Relative proportion of viral proteins in A/H3N2/Wisconsin stock*. Viral proteins were analysed by mass spectrometry proteomics, and the relative quantities of each protein was determined as described in the methods. Quantities are expressed as the percentage of the intensity of the top 3 peptides from each protein from 3 technical replicates.*
