# Supplementary material for: Immunopeptidomic analysis of influenza A virus infected human tissues identifies internal proteins as a rich source of HLA ligands
Source: PLoS Pathog. 2022 Jan 20;18(1):e1009894. doi: 10.1371/journal.ppat.1009894 (PMC8806059; doi:10.1371/journal.ppat.1009894)
Supplement: S3 Fig — A549 cells were infected at an MOI of 1.0 for 12 h, achieving >80% infection (Data are mean infection rates from replicate samples stained independently, n = 3 +/- SD). (PDF) [file ppat.1009894.s007.pdf]

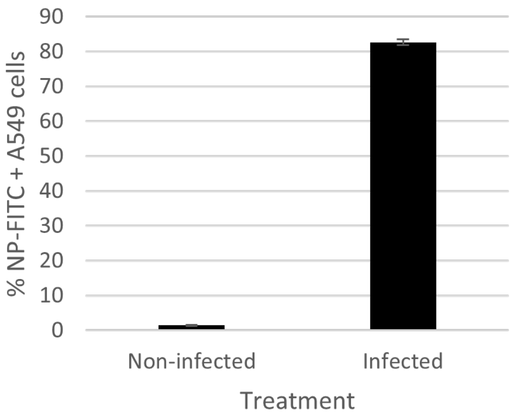

S3 Fig: Infection rates in A549 cells prior to DC engulfment. *A549 cells were infected at an MOI of 1.0 for 12 h, achieving >80% infection (Data are mean infection rates from replicate samples stained independently, n=3 +/- SD).*
