# Supplementary material for: Immunopeptidomic analysis of influenza A virus infected human tissues identifies internal proteins as a rich source of HLA ligands
Source: PLoS Pathog. 2022 Jan 20;18(1):e1009894. doi: 10.1371/journal.ppat.1009894 (PMC8806059; doi:10.1371/journal.ppat.1009894)
Supplement: S4 Fig — Lung tissue explants were infected with IFV-A ex-vivo and incubated post-infection for 24 h. Tissue samples were then enzymatically dispersed and the cells stained with monoclonal antibodies conjugated to cell-specific markers. Cell markers were used to identify (A) leukocytes (CD45-Horizon). (B) CD45+ cells were then gated to identify and exclude T cells (CD3-PECy7). (C) CD45+/CD3-/HLA-DR+ cells were macrophages. (D) CD45-CD326+ cells were identified as epithelial cells. (E) NP1/FITC staining was then used to identify infected macrophages (HLA-DR-APC/Cy7+/FITC+) and epithelial cells (F) (CD45-/CD326-PerCP/Cy5.5+/NP-FITC+). (PDF) [file ppat.1009894.s008.pdf]

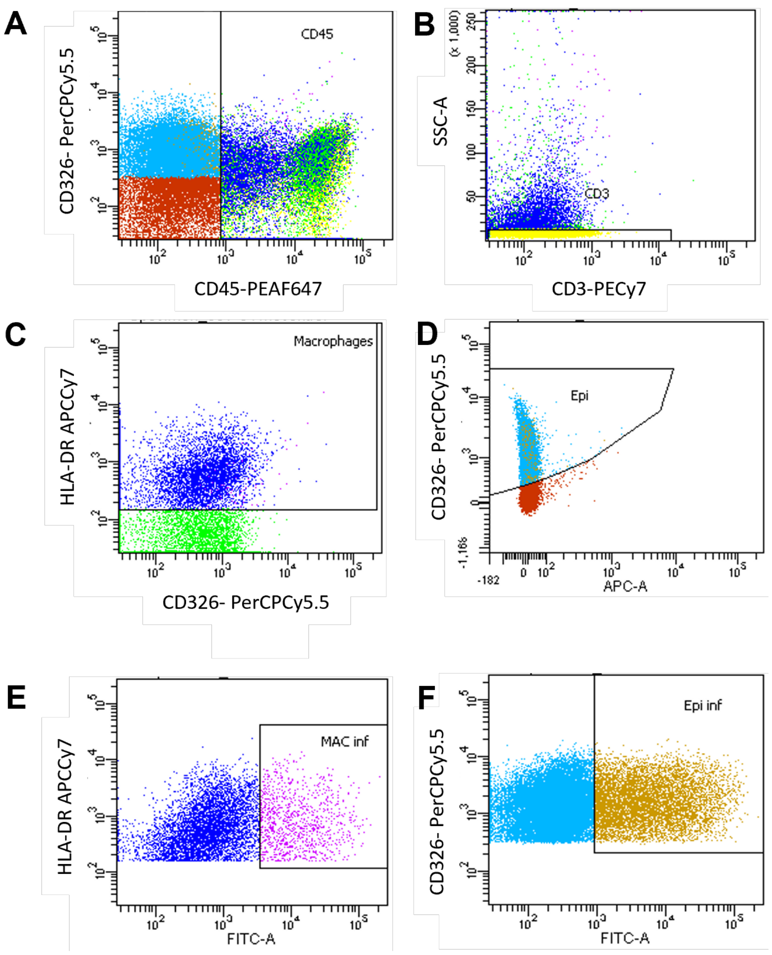

S4 Fig: Flow cytometric gating strategy for identification of influenza infection in lung tissue sample cell subsets. *Lung tissue explants were infected with IFV-A ex-vivo and incubated post-infection for 24 h. Tissue samples were then enzymatically dispersed and the cells stained with monoclonal antibodies conjugated to cell-specific markers. Cell markers were used to identify* ***(A)*** *leukocytes (CD45-Horizon).* ***(B)*** *CD45+ cells were then gated to identify and exclude T cells (CD3-PECy7).* ***(C)*** *CD45+/CD3-/HLA-DR+ cells were macrophages.* ***(D)*** *CD45-CD326+ cells were identified as epithelial cells.* ***(E)*** *NP1/FITC staining was then used to identify infected macrophages (HLA-DR-APC/Cy7+/FITC+) and epithelial cells* ***(F)*** *(CD45-/CD326-PerCP/Cy5.5+/NP-FITC+).*
