# Supplementary material for: Immunopeptidomic analysis of influenza A virus infected human tissues identifies internal proteins as a rich source of HLA ligands
Source: PLoS Pathog. 2022 Jan 20;18(1):e1009894. doi: 10.1371/journal.ppat.1009894 (PMC8806059; doi:10.1371/journal.ppat.1009894)
Supplement: Attachment — Submitted filename: plos-response-to-reviewers-letter-2020-12-02.pdf [file ppat.1009894.s009.pdf]

Centre for
Proteomic
Research

University of Southampton
Institute for Life Sciences
Building 85, University Rd
Southampton
SO17 1BJ
United Kingdom

Telephone +44(0)2380 59 5503

2nd December 2021

Dr Terri M Laufer
Guest Editor
PLOS Pathogens
plospathogens@plos.org

Dear Dr Laufer,

We are pleased to re-submit our manuscript entitled "**Immunopeptidomic analysis of influenza A virus infected human tissues identifies internal proteins as a rich source of HLA ligands**" along with our response to the reviewers' comments below.

Yours faithfully,

Dr Ben Nicholas

Line numbers refer to the clean document without tracked changes.

Responses to Major Issues:

Reviewer #1: The authors indicate the limitations of machine-based algorithms to predict the true efficacy of T cell epitopes and the need for in vitro and ex vivo models such as those they use in this manuscript. However, the authors did not compare the influenza peptide profiles they obtained from their experiments with those predicted by an algorithm for the given cells/HLA allotypes. To reiterate the importance of the experimental methods for determination of HLA ligands, it would be good to show whether all of the identified viral peptides were predicted by the algorithm, whether the top predictions from the algorithm were identified in vitro or not, if a higher frequency of predicted peptides were identified using one model or over the other, and if any similarities/differences could be identified between the peptides predicted by the algorithm and those which where or were not identified in vitro. As the authors did note in the manuscript, there are limitations in peptide identification by MS so this has to be taken in consideration when making inferences from the comparison between experimental and theoretical peptide identification, but the comparison should be made.

Response: We thank the reviewer for this request. To reiterate the importance of experimental methods for identifying HLA ligands we have amended the manuscript to highlight the experimentally observed peptides in the context of the thousands of possible predicted HLA ligands deriving from Wisconsin influenza proteins. The NetMHC predictions have been plotted and annotated with the positions of the observed peptides in the overall ranking. These analyses confirm that experimental observations are likely to be high affinity peptides, but that the algorithm ranking cannot determine which high affinity peptides will be observed.
This data is presented as an updated Figure 3 for the lung tissues, and additional panels have been incorporated in THP1 Figure 4F and MoDC Figure 5C. The corresponding text has been incorporated at lines 187-195, 264-278 and 337-339 for Figures 3,4 and 5 respectively.
The differences and similarities between the HLA-I peptides between the experimental models reflects the different or shared HLA allotypes, and their respective motifs. For example, both THP1 and P2 have the HLA-A*02:01 allotype and both present the AIMEKNIML peptide. We don’t observe any obvious differences in the frequency of HLA-I peptides between models, but the MoDC model does lead to observations of HLA-II peptides not seen in the other two models.

Reviewer #2: The authors mention alveolar macrophages in the abstract however, there is no direct experiment showing that dendritic cell engulf the apoptotic lung macrophages. Moreover, the role and the presence of interstitial macrophages should be also considered and dissected. Using the available FACS Aris instrument, the infected populations of antigen presenting cells should be determined.
The classical HLA alleles are the most polymorphic in the human population. The authors should involve more donors to support the conclusions of the manuscript.

Response: We mention in the abstract that, despite large numbers of infected alveolar macrophages in the ex-vivo lung tissue, we observed only a single HLA-II peptide by immunopeptidomics. Our conclusion in the text was that it is possible that infected macrophages do not contribute significantly to HLA-II presentation, at least in our ex-vivo system. This does not exclude the possibility that engulfment of infected macrophages might contribute to HLA-I presentation of viral peptides, however it would be difficult or impossible to differentiate between those and peptides presented by other infected cells such as epithelial cells. We have commented in the text that macrophages in the lung tissue were infected in significant numbers, and that we know that DCs are present in only very low numbers. We agree that a further analysis of APC subtypes and their infection rates would be very interesting and informative, but the gathering of this information would be outside the scope of this study as it would require greater quantities of lung material than those used in the manuscript.

Responses to Minor Issues:

Reviewer #1: In lines 172-176, the authors describe the number of peptides and proteins observed for each participant without context that these are host derived, as was done for THP1MΦ infection in lines 217-219. As the authors describe the virus-specific peptides in the lines immediately preceding the proteomic data, the natural assumption of the reader is that they are continuing to discuss virus-specific findings if clarification is not given.

Response: Thank you, we have clarified this text to distinguish between when we refer to host proteins and viral proteins on lines: 168-176.

Reviewer #1: Lines 185-187 would benefit from some clarification of the methods to identify allotypes and motifs. If I have understood correctly, you used unbiased cluster analysis of the observed host peptides from each sample to derive the 9-mer binding motifs for each HLA allotype (shown in Fig 2 C-E), and then used these binding motifs to match influenza peptides with HLA allotype (as indicated in Table 1). This is not directly clear from the description. To this effect, the figure legend for Fig. 2 C-E should just say "Class I HLA allotype 9-mer binding motifs derived from host immunopeptides using unsupervised clustering using MixMHCp."

Response: Thank you. We have made his amendment at lines 182-183.

Reviewer #1: In lines 248-249, the authors suggest that the majority of viral peptides were predicted to be strong binders to the HLA-B, however the majority of the peptides in Table 2 (including all HLA-B associated peptides) do not have predicted affinities to indicate the strength of their binding. Can the authors clarify how they are defining strong binders?

Response: Thank you for drawing our attention to this error. The correct THP1 allotype is HLA-B*15:11, which NetMHC cannot make predictions for. We have corrected the text and Table 2 to reflect that we assigned theses peptides as fitting the motif for HLA-B*15:11 and indicated where these ligands have previously been identified in the IEDB as ligands for HLA-I allotypes. We have also noted that in fact the other allotypes: HLA-A*02:01 and HLA-C*03:03 also have motifs that could fit these peptides. All three allotypes can bind peptides with hydrophobic amino acids and positions 2 and 9. These changes appear at lines 184-195.

Reviewer #1: In Table 2, there are no peptides in blue to indicate mis-matched aa sequences.

Response: Thank you for drawing our attention to this error. Table 2 has now been corrected and starts at line 268.

Reviewer #1: In Table 2, at first glance it looks like there are 2 unique peptides after Wisconsin infection, but this is because the NS1 peptide is listed twice to allow for reporting of the different predicted binding for the two virus strains. While this is understable, it would be helpful if those two peptides were at the very least listed directly after one another if not somehow noted to be the same.

Response: Thank you for drawing our attention to this issue. In Table 2 this is actually two different NS1 9-mer peptides: AIMEKNIML and AIMDKNIIL. But it was unclear due to the formatting. To make things clearer, all the peptide tables are now ordered by viral source protein and peptide length. Table 2 starts at line 268.

Reviewer #1: It is noted that nM is predicted affinity in Table S2, but this is not indicated in any of the main tables in the manuscript.

Response: Thank you for drawing our attention to this omission. A header note, “nM is NetMHC 4.0 predicted binding affinity.” has been added to each table. Lines 209, 271 and 335.

Reviewer #1: Table 3 has two “motif” columns.

Response: Thank you for drawing our attention to this error. This has been fixed. Table 3 starts at line 335.

Reviewer #1: Table S2 and Table S3 are switched, Table S2 should be the the summary of immunopeptidomes from cell lines, lung tissue, and DCs whereas Table S3 should be the immunopeptidome of A549 cells.

Response: Thank you for drawing our attention to this error. This has been fixed and Table S2 is the summary of immunopeptidomes, and the Table S3 the A549 immunopeptides. These tables are now in separate files.

Reviewer #1: There are some instances in which the wrong Table is referred to: Line 237 should be Table 2 and Line 292 should be Table S2.

Response: Thank you for drawing our attention to this error. References to the summary of immunopeptidomes now refer to Table S2. This table is now in a separate file.

Reviewer #1: There is an extra comma at the end of line 86 in the phrase “acquired infections, which also”

Response: Thank you for drawing our attention to this error, the extra comma has been removed at line 86.

Reviewer #1: In the methods for the phagocytosis assay, the authors mention monocyte generation media and DC activation supplement (line 566-567) but do not identify the components of these solutions or the source if purchased directly from a company.

Response: Thank you for drawing our attention to this issue. Lines 585-590 have been updated to include information about the specific kits purchased for the isolation and preparation of the MoDCs.

Reviewer #2: The purity of moDC cultures should be measured by the expression levels of CD1a(+), CD209(+), MHCII(high) and CD14(-).

Response: The aim of our DC work, which was intended as a proof-of-concept methodology for how HLA-II immunopeptides generated by in-vitro DCs might be identified, was to generate HLA-II viral immunopeptides using a controlled in-vitro system. We chose to use a proprietary kit which could be emulated elsewhere, accepting that such a methodology would generate DCs of a certain phenotype which, whilst not necessarily relevant to the phenotype of lung tissues per se, could still act as a guide to the likely products of DC antigen processing of infected cells. We used the Promocell DC generation kit to generate DCs. This kit is reported, when the supplied activation supplement is applied, to generate DCs with the following phenotype: CD14-, CD45+, CD83+. We have now included this information in the manuscript. We accept it would be desirable to analyse the phenotype of the cells used in the current study as confirmatory evidence of the expected phenotype.

However, expression of HLA-II is implicit in the immunopeptidomes we obtained from these cells, where the host-derived HLA-II peptides outnumber the HLA-I peptides, and was very much the key aim for this part of our study. We very much anticipate extending our current work to examine immunopeptidomes of other DC phenotypes,

although at the moment we have no evidence to suggest our findings would be significantly different.

Reviewer #2: Figure S1: The authors show one representative of three independent experiments. Please, incorporate a diagram showing the mean values +/- SD of the experiments.

Response: Thank you for this request. This information can be seen in Figure 2B at line 177.

Reviewer #2: Some minor mistyping issues are detected, please revise the manuscript to exclude such minor errors.

Response: Thank you for drawing our attention to this issue. We have made corrections throughout the revised manuscript.
